# Patient-Reported Outcomes and Return to Intended Oncologic Therapy After Colorectal Enhanced Recovery Pathway: The iCral3 Prospective Study

**DOI:** 10.1097/AS9.0000000000000267

**Published:** 2023-03-08

**Keywords:** colorectal surgery, ERAS, patient-reported outcomes, return to intended oncologic therapy

## Abstract

**Objective:**

To evaluate the influence of enhanced recovery pathway (ERP) on patient-reported outcome measures (PROMs) and return to intended oncologic therapy (RIOT) after colorectal surgery.

**Background:**

ERP improves early outcomes after colorectal surgery; however, little is known about its influence on PROMs and on RIOT.

**Methods:**

Prospective multicenter enrollment of patients who underwent colorectal resection with anastomosis was performed, recording variables related to patient-, institution-, procedure-level data, adherence to the ERP, and outcomes. The primary endpoints were PROMs (administered before surgery, at discharge, and 6 to 8 weeks after surgery) and RIOT after surgery for malignancy, defined as the intended oncologic treatment according to national guidelines and disease stage, administered within 8 weeks from the index operation, evaluated through multivariate regression models.

**Results:**

The study included 4529 patients, analyzed for PROMs, 1467 of which were analyzed for RIOT. Compared to their baseline preoperative values, all PROMs showed significant worsening at discharge and improvement at late evaluation. PROMs values at discharge and 6 to 8 weeks after surgery, adjusted through a generalized mixed regression model according to preoperative status and other variables, showed no association with ERP adherence rates. RIOT rates (overall 54.5%) were independently lower by aged > 69 years, ASA Class III, open surgery, and presence of major morbidity; conversely, they were independently higher after surgery performed in an institutional ERP center and by ERP adherence rates > median (69.2%).

**Conclusions:**

Adherence to the ERP had no effect on PROMs, whereas it independently influenced RIOT rates after surgery for colorectal cancer.

## Abstract 2

In this prospective multicenter study performed on 4529 patients who underwent colorectal resection, adherence to an enhanced recovery pathway showed no effect on patient-reported outcomes but independently influenced the return to intended oncologic therapy.

## BACKGROUND

Enhanced recovery pathway (ERP) is a multimodal and multifactorial approach to the optimization of perioperative management,^1,2^ designed to modify and improve the response to surgery-induced trauma based on a series of evidence-based items related to perioperative care.^3^ Several meta-analyses have shown a significant reduction in overall morbidity (OM) rates and length of stay after colorectal surgery.^4–6^ A significant dose-effect curve between adherence rate to ERP items and early outcomes has been demonstrated,^7–12^ and recent evidence derived from retrospective studies suggest that ERP may also offer a definite advantage over long-term survival after colorectal resection for malignancy.^13–15^ Apart from early outcomes, little is evident about patients’ experiences during the perioperative period. Measuring patient-reported outcomes address the gap in enhanced recovery assessment by incorporating patient-centered quality of life (QoL) into the global assessment of outcomes.^16^ At the same time, it is still unclear whether the ERP could improve the return to intended oncologic therapy (RIOT)^17^ after cancer surgery through the reduction in complications and poor performance status, both of which are associated with worse long-term oncologic outcomes.^18,19^ Therefore, the Italian ColoRectal Anastomotic Leakage study group planned this study (iCral3) to prospectively evaluate the impact of adherence to ERP items after colorectal resections on patient-reported outcome measures (PROMs) and RIOT after surgery for malignant disease.

## METHODS

### Study Design, Participants, and Setting

Prospective enrollment from November 2020 to October 2021 was carried out among 76 Italian surgical centers that voluntarily participated in iCral3. All patients undergoing elective or delayed urgency (>24 hours from admission) colorectal surgery with anastomosis were assessed for inclusion in a prospective database after obtaining written informed consent. Inclusion criteria were as follows: a) patients who underwent laparoscopic/robotic/open/converted colorectal resection with anastomosis, including planned Hartmann’s reversals; b) American Society of Anesthesiologists’ (ASA) class I, II, or III; c) elective or delayed urgency (>24 hours from admission) surgery; and d) patients’ written acceptance to be included in the study. The exclusion criteria were as follows: a) ASA class IV-V, b) emergent (≤24 hours from admission) surgery, c) pregnancy, and d) hyperthermic intraperitoneal chemotherapy for carcinomatosis.

According to the median number of cases enrolled during recruitment, each center was defined as high-volume (>median) or low-volume (≤median). The existence of an institutional ERP (having a locally implemented ERP team and protocol, supported by a specific resolution of the hospital/company strategic management) was declared by 48 out of 76 (63.1%) participating centers. All data of the included patients were prospectively uploaded into a web-based database via an electronic case report form, specifically designed for iCral3, protected by access credentials for each center/investigator.

### Clinical and Adherence Data

Continuous and discrete variables related to biometric data, patient-related and institution-related variables, indication and type of surgical procedure, and outcomes were recorded. Adherence to the 26 items of the ERP was measured for each single enrolled case upon criteria adapted from the 2018 ERAS Society^20^ and 2019 national^21^ guidelines (Table 1). Quality control of the data for consistency, plausibility, and completeness was performed on every single record by local investigators and subsequently validated by the study coordinator, resolving any discrepancies through strict cooperation.

**TABLE 1.** Definition and Adherence Criteria to Enhanced Recovery Pathway Items

ITEM	Adherence Criteria
Nutritional status screening	Patient submitted to nutritional status screening through Mini Nutritional Assessment Short Form (MNA-SF)
Nutritional prehabilitation	All patients showing MNA-SF < 12 (malnourished or suspect for malnutrition) and BMI > 30 (obesity) receive specific nutritional consultation
Physical prehabilitation	Patient receives a standard protocol of physical activity to be accomplished in the preoperative period; frail and limited motility patients are submitted to specific geriatrician/physiatrist consultation and personalized activity program
Psychologic prehabilitation	Patient and his familiars/caregivers are screened by the case manager; in case of anxiety/depression concerning diagnosis and related procedure psychologic consultation is warranted
Counseling	Patient and his familiars/caregivers receive full information and suggestions regarding the perioperative program from the surgeon, anesthesiologist, and case-manager
Preoperative immunonutrition	Patient is administered Impact Oral (Nestlè Health Science, Italy) 330 mL per os, 3 bricks per day during 5 d preceding surgery or 2 bricks per day during 7 d preceding surgery
Management of anemia	Patient with Hb concentration < 130 g/L for men and <120 g/L for nonpregnant women receive the correction of anemia before surgery preferably through intravenous iron preparations (ferric carboxymaltose) and blood transfusion(s) in strictly necessary cases
Antithrombotic prophylaxis	Patient receives graduate compression stockings and/or pneumatic compression device, together with prophylaxis with low molecular weight heparin during the perioperative period, to be extended up to 28 d after surgery in case of malignancy
Antibiotic prophylaxis	Patient is administered i.v. antibiotic 30 to 60 mins before incision, according to local protocols
No bowel preparation	No routine bowel preparation is used, except in case of the anticipated need for covering stoma
Oral carbohydrates load	Carbohydrates-rich beverage (12.5% maltodextrins) is given preoperatively (800 mL on the evening before surgery and 400 mL 2 to 3 hrs before surgery)
Preoperative fasting	Preoperative fasting is limited to 2 hrs for clear liquids (water, coffee, tea) and to 6 hrs for milk and solid food
No premedication	No long- or medium-action sedatives. Short and ultra-short-acting sedatives (eg, Lorazepam, Midazolam, Methohexital, Dexmedetomidine, Ketamine) are allowed before performing spinal, epidural, or loco-regional anesthesia
PONV prophylaxis	Postoperative nausea/vomiting prophylaxis is administered according to individual risk assessment (Apfel score) through a multimodal approach
Normothermia	Body temperature is monitored during surgery, utilizing fluid warmers and/or thermic blankets as necessary
Standard anesthetic protocol	General anesthesia through short-acting anesthetics, cerebral activity monitoring to enhance recovery and to reduce postoperative delirium, anesthesia level monitoring, and complete reversal of neuromuscular blockade
Intraoperative fluid management	Restrictive fluid therapy (defined as maintenance fluids at <2 mL/kg/h) or goal-oriented fluid therapy (stroke volume)
Multimodal analgesia	Use of more than 2 drugs or analgesia strategies (TAP-block or spinal anesthesia for minimally invasive surgery; thoracic epidural anesthesia for open surgery) to reduce the use of opiates
Minimally invasive surgery	Patient submitted to laparoscopic, robotic, or video-assisted surgery (conversions to open surgery included on an intention-to-treat basis)
No major opiates	Patient receives no major opiates in the postoperative period
No nasogastric tube	Nasogastric tube, if used, is removed at the end of surgery
No drain	No drain is placed in the abdominal cavity (pelvic drain allowed for pelvic surgery with low colorectal anastomosis)
Bladder catheter	Urinary catheter removed on POD 1 (up to POD 2 in case of pelvic surgery)
Early mobilization	Patient receives passive mobilization on POD 0, active mobilization on POD 1
Early oral feeding	Patient receives liquid oral diet starting 6 hrs after surgery and semisolid diet starting on POD 1
Pre-discharge check	Patient is checked just before discharge at home concerning adequate oral intake, bowel function, adequate pain control, active mobilization, no clinical/serological evidence of any postoperative complication, full agreement to go home

POD indicates postoperative day; PONV, postoperative nausea/vomiting.

During the perioperative period, patients were examined daily by local investigators, who were left free to decide on any complementary imaging and any further action according to their local criteria. Patients were followed up for a minimum of 8 weeks, during which data on all outcome measures and other study variables were collected.

### Outcomes

The primary endpoints were PROMs and RIOT.^17^ PROMs were administered to all enrolled patients 4 weeks to 1 day before the planned operation (preoperative), on the day of discharge (discharge), and 6 to 8 weeks after the operation (late), using the Euro-Quality of Life Group EQ-5D-5L,^22^ the MD Anderson Symptom Inventory for Gastrointestinal Surgery patients (MDASI-GI^23^), and the Functional Assessment of Cancer Therapy – Colorectal (FACT-C^24^) questionnaires. The EQ-5D-5L is a generic questionnaire on QoL divided into 2 sections: the EQ-5D index and the EQ-5D visual analog scale (VAS); the EQ-5D index assesses health status across 5 domains: anxiety/depression, mobility, pain/discomfort, self-care and usual activities, each scoring from 1 (worst) to 5 (best); EQ-5D VAS is a single 20 cm VAS with a range of 0 to 100, where 0 is the worst and 100 is the best imaginable health status. The EQ-5D-5L total scores ranged from 5 (worst) to 125 (best). The MDASI-GI is a specific questionnaire designed for evaluating digestive symptoms (18 questions) and the extent to which such symptoms interfere with daily activities (6 questions), each of which is presented as a VAS ranging from 0 (not present and/or not interfering at all) to 10 (as bad as you can imagine and/or completely interfering), with total scores ranging from 0 (best) to 240 (worst). The FACT-C is a specific colorectal cancer health questionnaire investigating physical, social/family, emotional, and functional well-being, with scores ranging from 0 (worst) to 136 (best).

RIOT rates, defined as the intended oncologic treatment according to the national guidelines for colorectal cancer and the disease stage^25^ administered within 8 weeks from the index operation, were recorded in all patients who underwent surgery for malignancy.

Secondary endpoints were anastomotic leakage (AL), defined and graded according to international^26^ and national^27^ consensus, OM (any adverse event, graded according to Clavien-Dindo^28,29^ and the Japanese Clinical Oncology Group-JCOG extended criteria^30^), major morbidity (MM, any adverse event grade > II), readmission, reoperation, and mortality rates. The overall postoperative length of stay (LOS) was calculated including any eventual readmission. All patients with a proximal diverting stoma at index operation underwent a routine check of anastomotic integrity through an intraluminal contrast exam (standard X-rays or CT scan), MRI, or direct endoscopic evaluation 3 to 8 weeks after the operation.

### Ethics and Dissemination

The study was conducted in accordance with the Declaration of Helsinki and the Guideline for Good Clinical Practice E6 (R2) principles. The study protocol was approved by the coordinating center ethics committee (Comitato Etico Regionale delle Marche – C.E.R.M. #2020/192, approved on 07/30/2020) and registered at ClinicalTrials.gov (NCT04397627). Thereafter, all participating centers obtained authorization from the local institutional review board. The study followed the Strengthening the Reporting of Observational Studies in Epidemiology reporting guidelines for cohort studies.^31^ Individual participant-level anonymized datasets were made available upon reasonable request by contacting the study coordinator.

### Statistical Analysis

All quantitative values are expressed as mean ± SD and 95% confidence intervals (95% CIs), median and interquartile range (IQR), and categorical data with percentage frequencies. For categorical data, the analysis included the use of cross-tabulation, chi-square, or Fisher’s exact test where indicated. Continuous or discrete variables were analyzed using Student’s 2-sided *t* test (allowing for heterogeneity of variances) or a non-parametric test (Mann-Whitney *U* test or Kruskal-Wallis test as indicated).

With the double intent of adjusting mean values for variables and identifying factors associated with PROMs, a generalized linear mixed regression model was used according to the “Setting International Standards in Analyzing Patient-Reported Outcomes and Quality of Life Endpoints Data Consortium” recommendations,^32^ where the dependent variables were PROMs at 2 different time-points (discharge and late) and independent items were all other variables, including baseline preoperative values for each PROM, AL, OM, MM, and reoperation. A generalized estimating equation^33^ was used to calculate model parameters to take into account the clustered, multicenter, nature of data. Each Beta coefficient measures the mean increment or decrement in PROMs values according to the presence of each independent item. To measure variable multicollinearity,^34^ the variance inflation factor was calculated using multiple linear regression for all the primary endpoints. No missing-value imputations were performed.

Quantitative variables such as age (years), operation length (minutes), and adherence rates (%) to the ERP were categorized according to their median values and/or their fourth centile. Other variables were categorized according to accepted predefined ranges: Mini Nutritional Assessment – Short Form (MNA-SF^35^) < 12, indicating potential malnutrition, or ≥12, indicating normal nutritional status; body mass index (Kg/m^2^) ≤25.0, 25.1 to 30.0, and >30.0. Surgical procedures were categorized as standard (anterior resection, right colectomy, left colectomy) versus non-standard (splenic flexure resection, transverse colectomy, Hartmann’s reversal, subtotal and total colectomy, and other) resections.^12^ The location of the tumor in case of malignancy was categorized as “right” (up to the transverse colon) or “left” (from the splenic flexure to the lower rectum).

A logistic regression analysis, excluding any variable with variance inflation factor ≥4, was used to assess associations between the examined variables and RIOT, presenting odds ratio (OR) and 95% CI.

For all statistical tests, the significance level was set at *P* < 0.05. All analyses were conducted using StatsDirect statistical software (StatsDirect Ltd., United Kingdom) and IBM SPSS Statistics for Windows, v.28.0 (Armonk, NY).

### Sample Size

Adherence to at least 70% of the ERP items was identified as a cutoff for significant improvement in outcomes,^9^ with a 2:1 expected ratio below:above this cutoff. Estimating a reduction of postoperative PROMs from preoperative baseline (1.0) at 0.7 for adherence above the cutoff and at 0.64 for adherence below the cutoff,^36^ alpha 0.04, beta 0.8, the required sample size was 2406 (802 cases above and 1604 cases below 70% adherence). Reported rates of failure to RIOT and ERP items adherence below or above 70% were 13% and 6.5%, respectively^19^; the required sample size for evaluation of RIOT was 885 (295 cases above and 590 cases below 70% adherence). Based on previous iCral observational studies on colorectal surgery in Italy,^12,37,38^ the expected ratio of malignant:benign indications to surgery was 70:30 (2100 resections for malignancy and 900 resections for benign disease based on 3000 expected cases).

## RESULTS

**FIGURE 1.**
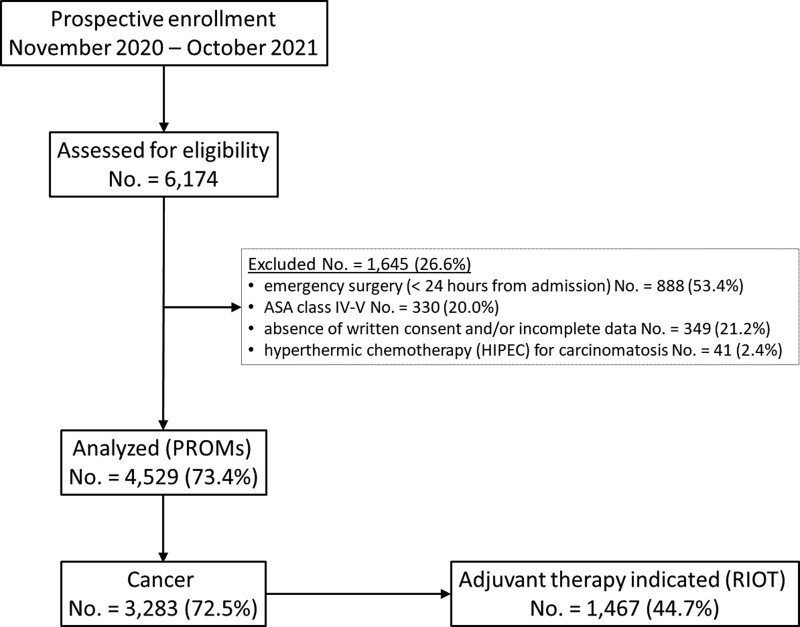
Study flowchart according to the Strengthening the Reporting of Observational Studies in Epidemiology (STROBE) Statement guidelines. ASA indicates American Society of Anesthesiologists; PROMs, patient-reported outcome measures; RIOT, return to intended oncologic therapy.

A total of 6174 potentially eligible cases were assessed, of which 4529 (73.3%) were included in the study and analyzed for PROMs (Fig. 1); surgery for malignancy was performed in 3283 cases (72.5%); indications for an adjuvant therapy according to disease stage and national guidelines^30^ were present in 1467 (44.7%) of these, representing the subpopulation analyzed for RIOT. Data regarding all the examined variables in both populations are reported in Supplemental Table 1, http://links.lww.com/AOSO/A218.

### Center Data

The median (IQR; range) number of assessed patients per single center was 66 (46 to 98; 15 to 362), while the median (IQR; range) number of included patients per single center was 44 (27 to 70; 12 to 344). The overall median (IQR) ERP adherence rate was 69.2% (53.8 to 80.8), being significantly higher in institutional ERP centers (median 73.1% vs 61.5%, *P* < 0.0001).

### Perioperative Outcomes

After a median (IQR; range) follow-up of 68 days (58 to 121; 0 to 402), 1726 adverse events (Table 2) were recorded in 1214 patients (OM rate 26.8%), of which 430 (24.9%) were Clavien-Dindo grade > II in 341 patients (MM rate 7.5%). There were 205 (4.5%) ALs, diagnosed after a median (IQR; range) of 5 (3 to 10; 1 to 99) days. AL diagnosis was established by intravenous contrast CT scan in 91 (44.4%), clinical criteria in 72 (35.2%), endoluminal contrast CT scan in 32 (15.6%), endoluminal contrast enema in 5 (2.4%) and gross findings at reoperation in the remaining 5 cases (2.4%). Regarding AL grading, a grade A leak was recorded in 42 cases (20.5%), grade B in 26 (12.7%), and grade C in the remaining 137 cases (66.8%). A proximal diverting stoma at index operation was performed in 292 (6.4%) cases; it was closed in all but 6 (2.0%) cases after a median (IQR; range) of 77 (54 to 154; 8 to 351) days.

**TABLE 2.** Adverse Events and Grading

Clavien-Dindo and JCOG Grade	I	II	IIIa	IIIb	IVa	IVb	Total
Anastomotic leakage	14	28	26	110	19	8	205
Surgical site infection	61	40	3	6	0	0	110
Abdominal collection/abscess	3	34	31	10	0	1	79
Intestinal obstruction	9	16	2	41	2	0	70
Anastomotic bleeding	33	20	15	1	0	0	69
Abdominal bleeding	4	23	4	22	2	0	55
Deep wound dehiscence	5	8	3	4	0	0	20
Small bowel perforation	0	0	0	12	2	0	14
Trocar/wound site bleeding	6	3	1	1	0	0	11
Anemia	23	192	1	0	0	0	216
Paralytic ileus	104	77	0	3	0	0	184
Fever	63	92	3	2	0	0	160
Pneumonia and pulmonary failure	9	38	5	1	9	4	66
Cardiac dysfunction and failure	10	35	1	2	6	8	62
Urinary retention	31	22	1	0	0	0	54
Urinary tract infection	6	22	1	0	0	0	29
Acute renal failure	16	19	3	0	5	0	43
Neurologic	22	13	0	1	0	1	37
DVT/pulmonary embolism	1	11	1	0	3	2	18
Acute peptic ulcer/erosive gastritis	0	1	1	1	0	0	3
Acute mesenteric ischemia	0	0	0	1	0	1	2
Other	115	67	16	10	5	6	219
Total	535	761	118	228	53	31	1,726

DVT indicates deep venous thrombosis.

There were 62 deaths (mortality rate 1.4%). Median overall LOS (IQR; range) was 6 (4 to 8; 0 to 91) days, with 174 re-admissions (3.8%) and 232 re-operations (5.1%).

### PROMs

**FIGURE 2.**
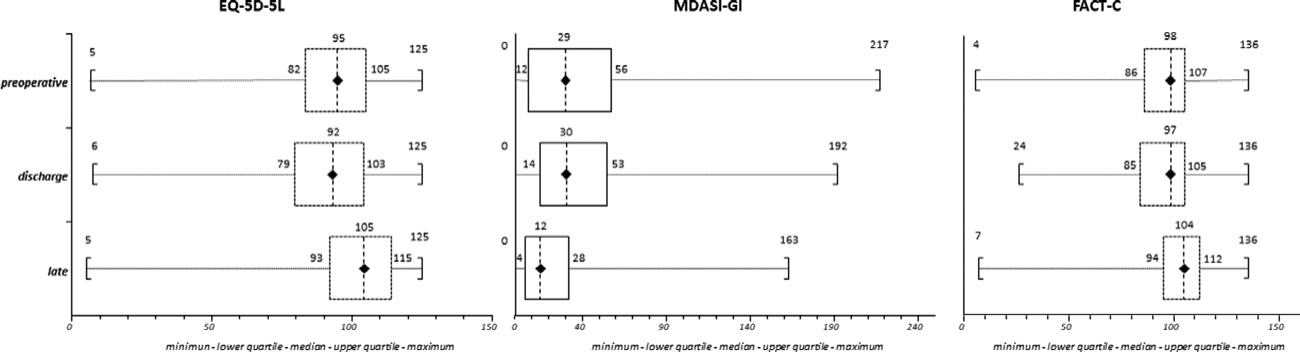
Box-whisker plots of unadjusted values of patient-reported outcome measures (PROMs); *P* < 0.0001 Kruskal-Wallis test; diamonds show the median values, boxes the IQR, and lines the range. EQ-5D-5L indicates Euro-Quality of Life Group EQ-5D-5L; FACT-C, Functional Assessment of Cancer Therapy – Colorectal; MDASI-GI, MD Anderson Symptom Inventory for Gastrointestinal Surgery patients.

Compliance with complete preoperative, discharge, and late evaluation QoL questionnaires varied from 88.1% to 95.5%; unadjusted PROMs values (Supplemental Table 2, http://links.lww.com/AOSO/A219) showed a significant worsening at discharge and a significant improvement beyond the preoperative baseline at late evaluation (Fig. 2), while their unadjusted values according to the median or fourth quartile of ERP adherence rates are shown in Table 3. After adjustment, no significant difference was detected in PROMs values according to the median or fourth quartile of ERP adherence rates (Table 4). The regression coefficients for the generalized mixed linear regression model considering ERP adherence rates below or above the median cutoff are reported in Table 5 (in Supplemental Table 3, http://links.lww.com/AOSO/A220, considering ERP adherence rates first to third vs fourth quartile). EQ-5D-5L adjusted values, both at discharge and at late evaluation, showed a significant negative association with ASA class III, presence of chronic renal failure, neoadjuvant therapy, OM, AL, length of procedure > 180 min, and, at discharge only, standard procedures. MDASI-GI adjusted values, both at discharge and at late evaluation, showed a significant negative association with female sex, neoadjuvant therapy, OM, AL, and length of procedure > 180 min. Similarly, FACT-C adjusted values at discharge and at late evaluation showed a significant negative association with ASA class III, presence of chronic renal failure, neoadjuvant therapy, elective admission, and AL. At the late evaluation, surgery for malignancy was independently linked to worst values for all PROMs.

**TABLE 3.** Unadjusted PROMs Values According to Median or 4th Quartile ERP Adherence Rates

	ERP Adherence (%)	Mean (SD)	*P*	ERP Adherence Centile	Mean (SD)	*P*
EQ_5D_5L preoperative	≤69.2	92.6 (21.0)	0.55	1st to 3rd	92.4 (20.7)	0.06
	>69.2	93.0 (19.9)		4th	93.7 (19.7)	
EQ_5D_5L at discharge	≤69.2	89.4 (19.3)	0.16	1st to 3rd	89.3 (19.4)	0.19
	>69.2	88.6 (19.8)		4th	88.4 (19.8)	
EQ_5D_5L late	≤69.2	102.0 (17.7)	0.007	1st to 3rd	101.6 (18.2)	0.17
	>69.2	100.5 (18.9)		4th	100.7 (18.8)	
MDASI-GI preoperative	≤69.2	40.2 (37.1)	0.02	1st to 3rd	39.9 (36.2)	0.008
	>69.2	37.6 (33.9)		4th	36.8 (34.3)	
MDASI-GI at discharge	≤69.2	39.3 (33.0)	0.01	1st to 3rd	38.8 (32.5)	0.053
	>69.2	36.9 (30.4)		4th	36.7 (30.2)	
MDASI-GI late	≤69.2	21.7 (25.4)	0.005	1st to 3rd	21.5 (25.1)	0.002
	>69.2	19.6 (24.0)		4th	18.8 (23.9)	
FACT-C preoperative	≤69.2	95.1 (17.0)	0.006	1st to 3rd	95.2 (16.7)	0.001
	>69.2	96.5 (15.8)		4th	97.1 (15.7)	
FACT-C at discharge	≤69.2	94.5 (15.0)	0.51	1st to 3rd	94.3 (15.1)	0.06
	>69.2	94.8 (15.0)		4th	95.3 (14.6)	
FACT-C late	≤69.2	100.2 (17.3)	0.25	1st to 3rd	100.2 (17.7)	0.18
	>69.2	100.9 (19.7)		4th	101.3 (20.1)	

EQ-5D-5L indicates Euro-Quality of Life Group EQ-5D-5L; FACT-C, Functional Assessment of Cancer Therapy – Colorectal; MDASI-GI, MD Anderson Symptom Inventory for Gastrointestinal Surgery patients; PROMs, patient-reported outcome measures.

**TABLE 4.** PROMs Values According to Median or 4th Quartile ERP Adherence Rates After Adjustment Through a Generalized Mixed Linear Regression Model

	ERP Adherence (%)	Mean (SD)	*P*	ERP Adherence Centile	Mean (SD)	*P*
EQ_5D_5L at discharge	≤69.2	80.3 (3.2)	0.81	1st to 3rd	80.1 (3.2)	0.79
	>69.2	79.2 (3.6)		4th	78.6 (3.4)	
EQ_5D_5L late	≤69.2	91.4 (2.3)	0.72	1st to 3rd	91.1 (2.4)	0.84
	>69.2	90.1 (2.7)		4th	90.3 (2.7)	
MDASI-GI at discharge	≤69.2	44.0 (5.1)	0.95	1st to 3rd	43.9 (5.0)	0.97
	>69.2	43.5 (5.4)		4th	43.6 (5.2)	
MDASI-GI late	≤69.2	31.7 (2.7)	0.81	1st to 3rd	31.5 (2.6)	0.82
	>69.2	30.8 (2.8)		4th	30.5 (3.1)	
FACT-C at discharge	≤69.2	92.8 (1.7)	0.83	1st to 3rd	92.6 (1.8)	0.95
	>69.2	92.2 (2.2)		4th	92.8 (2.1)	
FACT-C late	≤69.2	96.5 (2.2)	0.92	1st to 3rd	96.6 (2.2)	0.92
	>69.2	96.9 (2.8)		4th	97.0 (3.1)	

EQ-5D-5L indicates Euro-Quality of Life Group EQ-5D-5L; FACT-C, Functional Assessment of Cancer Therapy – Colorectal; MDASI-GI, MD Anderson Symptom Inventory for Gastrointestinal Surgery patients; PROMs, patient-reported outcome measures.

**TABLE 5.** Regression Coefficients After Generalized Linear Mixed Model Considering ERP Adherence Rates Below or Above the Median Cutoff

	EQ-5D-5L				MDASI-GI				FACT-C			
	Discharge		Late		Discharge		Late		Discharge		Late	
Variable	Beta ± SE	*P*	Beta ± SE	*P*	Beta ± SE	*P*	Beta ± SE	*P*	Beta ± SE	*P*	Beta ± SE	*P*
(Intercept)	46.91 ± 4.39	<0.001	74.17 ± 3.45	<0.001	16.72 ± 4.15	<0.001	4.06 ± 3.10	0.190	37.06 ± 3.79	<0.001	56.80 ± 7.05	<0.001
Age > 69 yrs	0.36 ± 0.69	0.604	−1.54 ± 0.46	<0.001	−3.59 ± 1.21	0.003	−0.80 ± 0.93	0.390	0.23 ± 0.44	0.602	−0.43 ± 0.73	0.561
Male sex	2.27 ± 0.54	<0.001	0.54 ± 0.61	0.384	−2.63 ± 0.91	0.004	−1.79 ± 0.73	0.014	0.57 ± 0.45	0.207	0.46 ± 0.57	0.422
ASA Class III	−1.92 ± 0.73	0.009	−2.85 ± 0.65	<0.001	1.46 ± 1.08	0.177	3.21 ± 0.94	<0.001	−1.65 ± 0.62	0.008	−1.76 ± 0.83	0.033
BMI > 30.0 Kg/m^2^	1.14 ± 0.82	0.168	−0.21 ± 0.77	0.782	−0.95 ± 1.12	0.396	0.13 ± 0.93	0.890	1.07 ± 0.48	0.026	−0.67 ± 0.90	0.457
BMI 25.1–30.0 Kg/m^2^	0.90 ± 0.65	0.164	0.10 ± 0.47	0.836	−1.67 ± 0.87	0.055	1.06 ± 0.73	0.144	0.11 ± 0.40	0.785	−0.96 ± 0.68	0.159
MNA-SF ≥ 12	0.22 ± 1.13	0.847	0.20 ± 0.99	0.839	−0.44 ± 2.07	0.831	0.37 ± 1.43	0.796	1.63 ± 0.71	0.023	0.03 ± 1.19	0.983
Diabetes	−1.43 ± 0.97	0.142	−0.62 ± 0.82	0.451	0.53 ± 1.44	0.712	0.87 ± 1.07	0.419	−0.54 ± 0.62	0.386	−1.31 ± 0.88	0.135
Chronic renal failure	−2.69 ± 1.24	0.030	−4.06 ± 1.35	0.003	2.79 ± 2.36	0.238	2.93 ± 1.71	0.087	−2.66 ± 1.02	0.009	−2.66 ± 1.47	0.070
Perioperative steroids	0.86 ± 2.44	0.726	−1.05 ± 1.92	0.582	−2.28 ± 3.15	0.468	1.88 ± 2.46	0.445	0.47 ± 1.21	0.698	0.50 ± 1.70	0.767
Neoadjuvant therapy	−4.04 ± 1.15	<0.001	−4.86 ± 1.07	<0.001	3.66 ± 1.88	0.052	6.05 ± 1.33	<0.001	−2.09 ± 0.71	0.003	−2.63 ± 0.98	0.007
Chronic liver disease	1.21 ± 2.06	0.557	−0.53 ± 1.92	0.784	−3.93 ± 2.36	0.238	0.41 ± 2.57	0.873	3.68 ± 1.71	0.031	−1.14 ± 1.91	0.550
Delayed urgency admission	1.84 ± 2.39	0.442	−0.03 ± 2.08	0.989	−7.57 ± 3.12	0.015	−2.57 ± 1.93	0.183	4.09 ± 1.54	0.008	3.70 ± 2.00	0.065
Preoperative blood transfusion(s)	−0.92 ± 1.06	0.389	−2.01 ± 1.09	0.064	−0.75 ± 1.48	0.611	1.71 ± 1.64	0.297	1.19 ± 0.87	0.174	1.54 ± 1.31	0.240
Intra-postoperative blood transfusion(s)	−2.26 ± 1.30	0.083	−0.47 ± 1.28	0.716	1.98 ± 2.02	0.328	1.12 ± 1.58	0.482	−0.59 ± 1.03	0.564	0.17 ± 1.18	0.885
Procedure length > 180 mins	−2.12 ± 0.87	0.015	−1.27 ± 0.69	0.068	4.39 ± 1.44	0.002	2.45 ± 1.03	0.017	−1.21 ± 0.61	0.046	−0.76 ± 0.72	0.291
Associated procedures	−2.75 ± 1.52	0.070	−1.35 ± 1.72	0.431	5.00 ± 2.62	0.057	1.44 ± 1.69	0.392	−1.45 ± 0.92	0.117	−1.82 ± 1.28	0.154
Minimally invasive surgery	−0.57 ± 1.31	0.664	0.92 ± 0.97	0.347	2.68 ± 2.42	0.268	0.38 ± 1.62	0.813	0.66 ± 0.85	0.436	0.71 ± 1.18	0.547
ERP adherence rate > median	−1.14 ± 1.66	0.491	−1.31 ± 1.36	0.334	−0.48 ± 3.21	0.880	−0.96 ± 1.56	0.541	−0.59 ± 1.19	0.619	0.37 ± 1.83	0.841
High-volume center	1.08 ± 1.60	0.503	0.07 ± 1.26	0.957	−4.99 ± 3.36	0.138	−1.70 ± 2.14	0.426	2.22 ± 1.07	0.039	0.57 ± 1.73	0.742
Institutional ERP center	−1.04 ± 2.08	0.617	−2.51 ± 1.65	0.130	1.33 ± 4.67	0.776	−1.27 ± 2.52	0.613	−1.69 ± 1.34	0.207	−1.89 ± 1.73	0.274
Anastomotic leakage	−6.49 ± 1.99	<0.001	−4.78 ± 1.44	<0.001	7.48 ± 3.93	0.057	4.44 ± 2.50	0.076	−4.57 ± 1.37	<0.001	−5.01 ± 1.79	0.005
Overall morbidity	−1.60 ± 0.69	0.020	−1.42 ± 0.56	0.011	2.95 ± 1.16	0.011	1.75 ± 0.73	0.018	−0.83 ± 0.48	0.088	−1.11 ± 0.61	0.068
Major morbidity	−1.75 ± 2.06	0.396	−2.32 ± 1.37	0.090	−0.66 ± 2.90	0.819	2.99 ± 2.84	0.294	−1.49 ± 1.30	0.253	−2.27 ± 1.87	0.226
Reoperation	−3.68 ± 2.61	0.158	−1.99 ± 2.04	0.330	7.20 ± 3.73	0.054	1.52 ± 3.86	0.694	−0.70 ± 1.65	0.672	2.06 ± 1.90	0.280
Standard procedure	−2.54 ± 1.07	0.018	−0.03 ± 0.84	0.973	2.48 ± 1.66	0.135	0.08 ± 1.06	0.939	−0.70 ± 1.65	0.672	0.77 ± 1.01	0.445
Surgery for malignancy	0.79 ± 1.16	0.497	−3.66 ± 1.22	0.003	−0.61 ± 2.06	0.768	3.70 ± 1.53	0.015	−0.30 ± 0.61	0.620	−3.47 ± 0.99	<0.001
PROMs preoperative values	0.50 ± 0.04	<0.001	0.38 ± 0.04	<0.001	0.50 ± 0.04	<0.001	0.33 ± 0.03	<0.001	0.61 ± 0.03	<0.001	0.50 ± 0.07	<0.001
(Scale)	256.10		248.41		665.38		457.95		126.28		258.96	

ASA indicates American Society of Anesthesiologists; BMI, body mass index; BMI, body mass index; EQ-5D-5L, Euro-Quality of Life Group EQ-5D-5L; ERP, enhanced recovery pathway; FACT-C, Functional Assessment of Cancer Therapy – Colorectal; MDASI-GI, MD Anderson Symptom Inventory for Gastrointestinal Surgery patients; MNA-SF, mini nutritional assessment short form; PROMs, patient-reported outcome measures.

### RIOT

**FIGURE 3.**
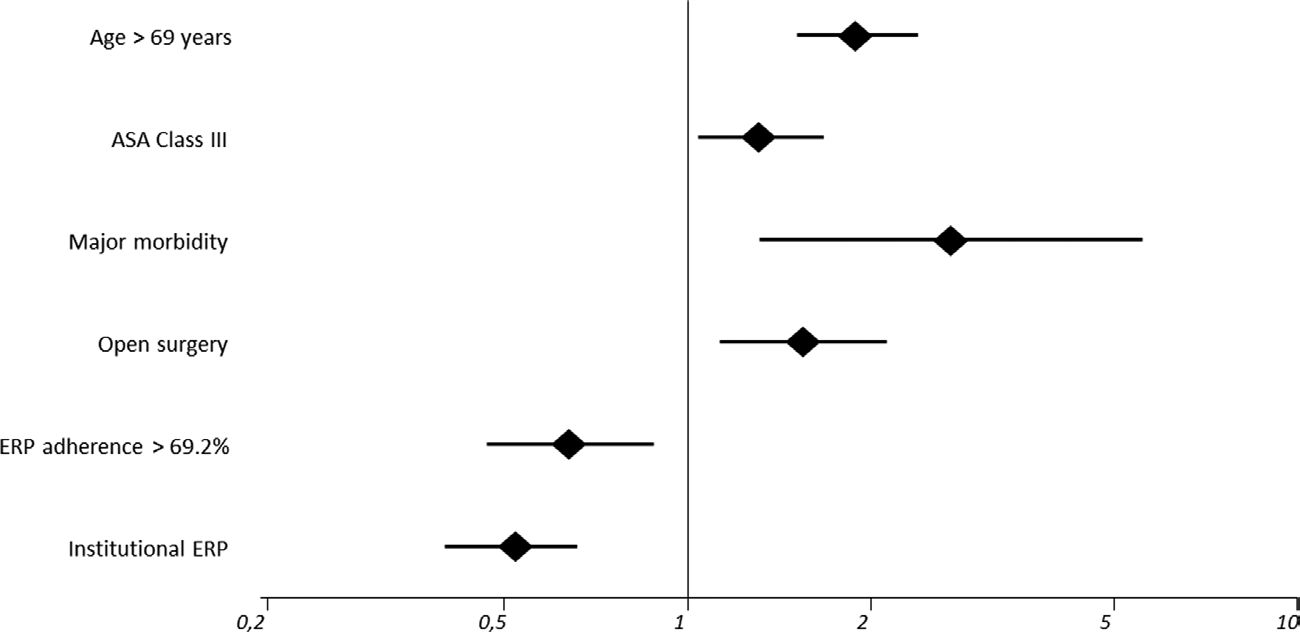
Forest plot (log scale) of independent variables for return to intended oncologic therapy (RIOT); diamonds show ORs, and lines show 95% CIs. ASA indicates American Society of Anesthesiologists; ERP, enhanced recovery pathway.

RIOT was recorded in 800 out of 1467 cases (54.5%); an adjuvant treatment was administered beyond 8 weeks after the index operation in 400 cases (27.3%) and not administered at all in the remaining 267 cases (18.2%). After the multivariate analysis (Table 6 and Fig. 3), RIOT was independently lower in patients aged > 69 years (OR 1.89; 95% CI 1.51 to 2.38), ASA Class III (OR 1.31; 95% CI 1.03 to 1.67), major morbidity (OR 2.70; 95% CI 1.31 to 5.55) and open surgery (OR 1.55; 95% CI 1.13 to 2.12). Conversely, it was independently higher when surgery was performed in an institutional ERP center (OR 0.52; 95% CI 0.40 to 0.66) and by adherence to ERP above the median (OR 0.64; 95% CI 0.47 to 0.88).

**TABLE 6.** Univariate and Multivariate Analyses for the Return to Intended Oncologic Therapy (RIOT)

		Univariate analysis				Multivariate analysis					
Variable	Pattern	RIOT	NO RIOT	%	*P*	VIF	Beta	Beta SE	OR	95% CI	*P*
Age (yrs)	≤69	474	281	62.8	<0.0001	1.11	-0.64	-5.53	0.53	0.42-0.66	<0.0001
	>69	326	386	45.8							
Gender	Female	355	275	56.3	0.246						
	Male	445	392	53.2							
ASA class	I–II	568	391	59.2	<0.0001	1.14	-0.27	-2.22	0.76	0.60-0.97	0.026
	III	232	276	45.7							
BMI (Kg/m^2^)	<25	394	282	58.3	0.021	1.04	-0.11	-1.38	0.89	0.76-1.05	0.167
	25–30	296	290	50.5							
	>30	110	95	53.7							
Diabetes	Yes	110	101	52.1	0.449						
	No	690	566	54.9							
Chronic renal failure	Yes	30	30	50.0	0.471						
	No	770	637	54.7							
Dialysis	Yes	2	1	66.7	>0.999						
	No	798	666	54.5							
Perioperative steroids	Yes	6	10	37.5	0.209						
	No	794	657	54.7							
Neoadjuvant therapy	Yes	93	90	50.8	0.281						
	No	707	577	55.1							
Chronic liver disease	Yes	10	10	50.0	0.682						
	No	790	657	54.6							
MNA-SF	<12	306	306	50.0	0.003						
	≥12	494	361	57.8		1.07	-0. 01	-0.11	0.99	0.78-1.24	0.910
Preoperative blood transfusion(s)	Yes	57	51	52.8	0.779						
	No	743	616	54.7							
Intra- and postoperative blood transfusion(s)	Yes	48	63	43.2							
	No	752	604	55.5	0.017	1.06	-0.12	-0.57	0.88	0.58-1.35	0.565
Center volume (No. of cases)	Low (≤44)	195	160	54.9	0.911						
	High (>44)	605	507	54.4							
Institutional ERP	Yes	325	203	61.5	<0.0001	1.15	-0.66	-5.32	0.52	0.40-0.66	<0.0001
	No	475	464	50.6							
Hospital type	District/regional	346	289	54.5							
	Academic/teaching	258	245	51.3	0.0716						
	Metropolitan	196	134	57.0							
Center type	General surgery	671	547	55.5							
	Oncologic surgery	73	69	51.4	0.631						
	Colorectal surgery	56	51	52.3							
Admission	Elective	757	611	55.3		1.06	-0.14	-0.62	0.87	0.56-1.35	0.536
	Urgent	43	56	43.4	0.028						
Tumor location	Right	361	314	53.5	0.487						
	Left	439	353	55.4							
Procedure	Standard	714	576	55.3	0.106						
	Non-standard	86	91	48.6							
Procedure length	≤180 mins	423	318	57.1	0.053						
	>180 mins	377	349	51.9							
Associated procedures	Yes	142	131	52.0	0.354						
	No	658	536	55.1							
Minimally invasive surgery	Yes	706	530	57.1							
	No	94	137	40.7	<0.0001	1.11	0.44	2.75	1.55	1.13-2.12	0.006
ERP adherence rate (%)	>69.2	408	251	61.9							
	≤69.2	392	416	48.5	<0.0001	1.49	0.50	3.64	1.55	1.13-2.12	0.0003
ERP adherence centile	4th	254	154	62.2							
	1st to 3rd	546	513	51.6	0.0003	1.54	0.31	1.73	1.36	0.96-1.92	0.083
Anastomotic leakage	Yes	18	50	26.5							
	No	782	617	55.9	<0.0001	1.71	-0.61	-1.67	0.54	0.26-1.11	0.095
Overall morbidity	Yes	189	207	47.7	0.002	1.14	0.011	0.082	0.99	0.76-1.28	0.997
	No	611	460	57.1							
Major morbidity	Yes	35	87	28.7							
	No	765	580	56.9	<0.0001	2.78	-0.99	-2.7	0.37	0.18-0.76	0.007
Reoperation	Yes	24	53	31.2	<0.0001	2.63	0.20	0.45	1.22	0.51-2.91	0.651
	No	776	614	55.8							

ASA indicates American Society of Anesthesiologists; ERP, enhanced recovery pathway; MNA-SF, Mini Nutritional Assessment Short Form; OR, odds ratio; SE, standard error; VIF, variance inflation factor.

## DISCUSSION

This prospective multicenter observational study investigated the effects of the ERAS program on PROMs and RIOT after elective colorectal surgery in more than 4000 patients enrolled over a 12-months period in 76 Italian surgical centers, without any limitation concerning the presence of an institutional ERP or center caseload.

During the design of the current study, we carefully screened the discriminant properties of existing PROMs, being aware that there is still very limited evidence supporting the measurement properties of existing PROMs used to evaluate recovery after abdominal surgery,^39^ many of which fail to detect even obvious differences, such as those related to the type of surgical approach.^40^ Moreover, a previous prospective series of 100 cases using a different set of PROMs^36^ failed to detect any influence of ERP adherence on late postoperative recovery, hypothesizing either a lack of association or a lack of PROMs’ ability to detect any difference. Therefore, we decided to use 3 different questionnaires: EQ-5D-5L was chosen as a generic QoL index because of its worldwide availability (>130 languages), low (<5 minutes) time requirement, and great discriminatory power; MDASI-GI as a digestive disease-specific instrument because it is concise, stable and comprehensive, using simple and familiar 0 to 10 scales; and FACT-C as a colorectal cancer-specific index because of its disease-specific discriminatory power.^41,42^ As a matter of fact, the raw values of all these 3 QoL instruments showed a parallel and significant worsening at discharge and improvement at late (6 to 8 weeks after surgery) evaluation compared to their preoperative values (Fig. 2), with high compliance rates ranging from 88.1 to 95.5% of cases (Supplemental Table 2, http://links.lww.com/AOSO/A219). This finding confirms the reliability of the PROMs set used in the current study in recording the strong influence of major abdominal surgery on patients’ perceived QoL and functional status, and that postoperative recovery takes time beyond hospital discharge.^43^ At the same time, raw PROMs values showed little or no significant association with ERP adherence rates, with only MDASI-GI scores significantly improved both at discharge and at late evaluation according to the fourth centile (Table 3). Once raw data were adjusted through a generalized linear mixed regression model accounting for baseline PROMs values and all other variables considered in the study,^32^ it appeared that ERP adherence rates had no significant effect on patient-reported outcomes (Tables 4 and 5). This finding may stem, as already recorded,^36,39^ from the inability of the current PROMs set to detect any difference. Considering that neoadjuvant treatments, surgery for malignancy, and the occurrence of morbidity and AL showed an independent negative association with nearly all PROMs and time spans used in the current analysis (Table 5), this finding is more likely related to the independent detrimental effect of cancer and postoperative complications on self-reported recovery after colorectal surgery.

Failure to detect any independent effect of ERP adherence on patients’ perceived QoL may appear as a negative result of the study; on the other hand, it could be intended as a starting point or as the end of the beginning in the clinical research area dealing with the measurement of patient-reported outcomes in colorectal surgery; all the clinicians (surgeons as well) involved in the perioperative journey of the patient should, on the one hand, continue to struggle to improve time-honored outcome measures such as morbidity, mortality and postoperative LOS, and, at the same time, move away from them and forward to the individuation of an entirely new set of PROMs, able to address patients’ still unmet needs.^44,45^

RIOT rates were defined and calculated as the actual number of patients receiving the indicated adjuvant therapy within 8 weeks from the index operation, as timely initiation is linked to improved long-term results.^46^ The overall rate of 54.5% recorded in this study is within the literature range,^47^ and it was independently lower in older and comorbid patients, open surgery, and in presence of major morbidity (Table 6 and Fig. 3). This is not surprising, since these factors are commonly reported as the main determinants of failure of RIOT after surgery for colorectal cancer.^48,49^ Both the presence of an institutional ERP and its adherence rate beyond the median cutoff (69.2%) resulted in independent protective factors. While a previous multivariate analysis of a smaller retrospective cohort comparing pre- and post-ERP implementation identified significantly higher RIOT rates in the post-implementation group,^49^ the current study is, to our knowledge, the first to identify an independent role of ERP adherence and institutionalization on RIOT rates in a larger and multicenter prospective cohort. This effect could partially explain the impact of ERP adherence on long-term oncologic outcomes recorded in previous retrospective series.^13–15^ For this purpose, a 3- and 5-year follow-up of all oncologic patients enrolled in the present cohort has already been planned.

The median number of enrolled patients per center in the present study (No.= 44) was significantly lower than that (No.= 82) recorded in the previous iCral2 study,^12^ and while OM (26.8%) and AL (4.5%) rates recorded in the present study were similar to those recorded in the previous study, MM (7.5%) and mortality (1.4%) rates were somewhat higher. The lower accrual rate in the present study is probably due to 2 main factors. First, the world coronavirus pandemic had a deep impact on the reduction of elective surgical activities^50^; second, the inclusion of PROMs in the investigation protocol led to a higher number of exclusions due to consent denial and/or incomplete data (Fig. 1). Anyway, this prospective cohort was more representative (inclusion rate 73.3%) than that of the previous study (inclusion rate 57.8%), probably because proximal diverting stoma and delayed urgency cases were included. These cases may be responsible for the higher MM and mortality rates compared to those in the previous study.

This study has several strengths: it represents, by far, the largest multicenter prospective investigation on some of the currently available PROMs after colorectal surgery; it was performed in a well-defined time-lapse in a large number of centers representing a wide sample of surgical units performing colorectal surgery in Italy; the prospective design of the study allowed the measurement of outcomes through adherence to ERP items in all the enrolled cases, responding to clear and sheer compliance criteria. Its main limitation is the potential for residual, measured, and unmeasured confounding intrinsic to any observational study. Moreover, although data quality control was performed and repeated at various levels, we cannot exclude any measurement error from the participating investigators.

In conclusion, this study brings 2 important findings: on one side, the confirmation that every effort should be made to improve ERP adherence rates since this could improve long-term oncologic outcomes through its independent boosting effect on the timely RIOT; on the other side, the consciousness that this is still not sufficient to significantly improve patients’ self-perceived QoL during their perioperative journey. Further clinical research to cope with this caregiving shortcoming should be strongly encouraged.

## ACKNOWLEDGMENTS

iCral3 study group investigators with shared authorship are: Marco Catarci, MD, FACS (Study coordinator) (General Surgery Unit, Sandro Pertini Hospital, ASL Roma 2), Giacomo Ruffo, MD (General Surgery Unit, IRCCS Sacro Cuore Don Calabria Hospital, Negrar di Valpolicella (VR)), Massimo Giuseppe Viola, MD (General Surgery Unit, Cardinale G. Panico Hospital, Tricase (LE)), Ferdinando Ficari, MD (General Surgery and IBD Unit, Careggi University Hospital, Firenze), Paolo Delrio, MD (Colorectal Surgical Oncology, Istituto Nazionale per lo Studio e la Cura dei Tumori, “Fondazione Giovanni Pascale IRCCSItalia”, Napoli), Felice Pirozzi, MD (General Surgery Unit, ASL Napoli 2 Nord, Pozzuoli (NA)), Felice Borghi, MD (General & Oncologic Surgery Unit, Department of Surgery, Santa Croce e Carle Hospital, Cuneo), Raffaele De Luca, MD (Department of Surgical Oncology, IRCCS Istituto Tumori “Giovanni Paolo II”, Bari), Alberto Patriti, MD (Department of Surgery, Marche Nord Hospital, Pesaro e Fano (PU)), Gianluca Garulli, MD (General Surgery Unit, Infermi Hospital, Rimini), Walter Siquini, MD (General Surgery Unit, S. Lucia Hospital, Macerata), Stefano D’Ugo, MD, PhD, FEBS, FACS (General Surgery Unit, “V. Fazzi” Hospital, Lecce), Stefano Scabini, MD (General & Oncologic Surgery Unit, IRCCS “San Martino” National Cancer Center, Genova), Marco Caricato, MD, FACS (Colorectal Surgery Unit, Policlinico Campus BioMedico, Roma), Giusto Pignata, MD (2nd General Surgery Unit 2, Spedali Civili di Brescia), Andrea Liverani, MD (General Surgery Unit, Regina Apostolorum Hospital, Albano Laziale (RM)), Roberto Campagnacci, MD (General Surgery Unit, “C. Urbani” Hospital, Jesi (AN)), Pierluigi Marini, MD (General & Emergency Surgery Unit, San Camillo-Forlanini Hospital, Roma), Ugo Elmore, MD (Department of Gastrointestinal Surgery Unit, San Raffaele Research Hospital and “Vita-Salute” San Raffaele University, Milano), Francesco Corcione, MD (Minimally Invasive General and Oncologic and Surgery Unit, “Federico II” University, Napoli), Roberto Santoro, MD (General Oncologic Surgery Unit, Belcolle Hospital, Viterbo), Massimo Carlini, MD, FACS (General Surgery Unit, S. Eugenio Hospital, ASL Roma 2), Antonio Giuliani, MD (General Surgery Unit, S. Carlo Hospital, Potenza), Mario Sorrentino, MD (General Surgery Unit, Latisana-Palmanova Hospital, Friuli Centrale University (UD)), Giovanni Ferrari, MD (General Oncologic and Mininvasive Surgery Unit, Great Metropolitan Niguarda Hospital, Milano), Gianandrea Baldazzi, MD (General Surgery Unit, ASST Ovest Milanese, Nuovo Ospedale di Legnano, Legnano (MI)), Alberto Di Leo, MD (General and Minimally Invasive Surgery Unit, San Camillo Hospital, Trento), Augusto Verzelli, MD (General Surgery Unit, Profili Hospital, Fabriano (AN)), Giuseppe Sica, MD (Minimally Invasive Surgery Unit, Policlinico Tor Vergata University Hospital, Roma), Stefano Rausei, MD (General Surgery Unit, Gallarate Hospital (VA)), Davide Cavaliere, MD (General & Oncologic Surgery Unit, AUSL Romagna, Forlì (FC)), Gian Luca Baiocchi, MD, FACS (General Surgery Unit 3, Department of Clinical and Experimental Sciences, University of Brescia), Marco Milone, MD (General & Endoscopic Surgery Unit, “Federico II” University, Napoli), Giovanni Ciaccio, MD (General Surgery Unit, S. Elia Hospital, Caltanissetta), Giovanni Domenico Tebala, MD, FACS, FRCS (General Surgery Unit, S. Maria Hospital, Terni), Marco Scatizzi, MD (General Surgery Unit, Santa Maria Annunziata Hospital, Firenze), Luigi Boni, MD, FACS (General Surgery Unit, Fondazione IRCCS Ca’ Granda, Policlinico Maggiore Hospital, Milano), Stefano Mancini, MD (General & Oncologic Surgery Unit, San Filippo Neri Hospital, ASL Roma 1), Mario Guerrieri, MD (Surgical Clinic, Torrette Hospital, University of Ancona), Roberto Persiani, MD (General Surgery Unit, Fondazione Policlinico Universitario Agostino Gemelli IRCCS, Roma), Andrea Lucchi, MD, FACS (General Surgery Unit, “Ceccarini” Hospital, Riccione (RN)), Dario Parini, MD (General Surgery Unit, S. Maria della Misericordia Hospital, Rovigo), Antonino Spinelli, MD (Department of Biomedical Sciences, Humanitas University, Pieve Emanuele (MI) and IRCCS Humanitas Research Hospital, Rozzano (MI)), Michele Genna, MD (General Surgery Unit, University Hospital, Verona), Vincenzo Bottino, MD (General & Oncologic Surgery Unit, Evangelico Betania Hospital, Napoli), Andrea Coratti, MD (General and Emergency Surgery Unit, Misericordia Hospital, Grosseto), Dario Scala, MD (Abdominal Oncologic Surgery Unit, IRCCS CROB Basilicata Referral Cancer Center, Rionero in Vulture (PZ)), Andrea Muratore, MD (General Surgery Unit, “E. Agnelli” Hospital, Pinerolo (TO)), Maurizio Pavanello, MD (General Surgery Unit, AULSS2 Marca Trevigiana, Conegliano Veneto (TV)), Umberto Rivolta, MD (General Surgery Unit, Fornaroli Hospital, ASST Ovest Milanese, Magenta (MI)), Micaela Piccoli, MD, FACS (General Surgery Unit, Civil Hospital, Baggiovara (MO)), Carlo Talarico, MD (General Surgery Unit, Villa dei Gerani Hospital, Vibo Valentia (VV)), Alessandro Carrara, MD (1st General Surgery Unit, S. Chiara Hospital, Trento), Stefano Guadagni, MD (General Surgery Unit, University of L’Aquila), Mauro Totis, MD (Colorectal Surgery Unit, San Gerardo Hospital, ASST Monza), Franco Roviello, MD (General & oncologic Surgery Unit, AOU Senese, Siena), Alessandro Anastasi, MD (General Surgery Unit, San Giovanni di Dio Hospital, Firenze), Gianluca Guercioni, MD (General Surgery Unit, “C. e G. Mazzoni” Hospital, Ascoli Piceno), Giuseppe Maria Ettorre, MD (General & Transplant Surgery Unit, San Camillo-Forlanini Hospital, Roma), Mauro Montuori, MD (General & Mininvasive Surgery Unit, S. Pietro Hospital, Ponte San Pietro (BG)), Pierpaolo Mariani, MD (General Surgery Unit, Pesenti Fenaroli Hospital, Alzano Lombardo (BG)), Nicolò de Manzini, MD (Surgical Clinic, University of Trieste), Annibale Donini, MD (General & Emergency Surgery Unit, University of Perugia), Mariano Fortunato Armellino, MD (General & Emergency Surgery Unit, S. Giovanni di Dio e Ruggi d’Aragona Hospital, Salerno), Lucio Taglietti, MD (General Surgery Unit, ASST Valcamonica, Esine (BS)), Gabriele Anania, MD (General & Laparoscopic Surgery Unit, University Hospital, Ferrara), Mariantonietta Di Cosmo, MD (General & Upper GI Surgery Unit, University Hospital, Verona), Carlo Vittorio Feo, MD (General Surgery Unit, Delta Hospital, Lagosanto (FE)), Paolo Millo, MD (General Surgery Unit, “U. Parini” Regional Hospital, Aosta), Corrado Pedrazzani, MD (General & HPB Surgery Unit, University Hospital, Verona), Silvio Guerriero, MD (General Surgery Unit, “A. Murri” Hospital, Fermo), Andrea Costanzi, MD (General Surgery Unit, S. Leopoldo Hospital, Merate (LC)), Nereo Vettoretto, MD (General Surgery Unit, Spedali Civili of Brescia, Montichiari (BS)), Federico Marchesi, MD (Surgical Clinic, University of Parma), Massimo Basti, MD (General Surgery Unit, Spirito Santo Hospital, Pescara), Graziano Longo, MD (General Surgery Unit, Policlinico Casilino, Roma), Moreno Cicetti, MD (General Surgery Unit, S. Maria della Misericordia Hospital, Urbino (PU)), Paolo Ciano, MD (General Surgery Unit, Sandro Pertini Hospital, ASL Roma 2), Michele Benedetti, MD (General Surgery Unit, Sandro Pertini Hospital, ASL Roma 2), Leonardo Antonio Montemurro, MD (General Surgery Unit, Sandro Pertini Hospital, ASL Roma 2), Maria Sole Mattei, MD (General Surgery Unit, Sandro Pertini Hospital, ASL Roma 2), Elena Belloni, MD (General Surgery Unit, Sandro Pertini Hospital, ASL Roma 2), Elisa Bertocchi, MD (General Surgery Unit, IRCCS Sacro Cuore Don Calabria Hospital, Negrar di Valpolicella (VR)), Gaia Masini, MD (General Surgery Unit, IRCCS Sacro Cuore Don Calabria Hospital, Negrar di Valpolicella (VR)), Amedeo Altamura, MD (General Surgery Unit, Cardinale G. Panico Hospital, Tricase (LE)), Francesco Rubichi, MD (General Surgery Unit, Cardinale G. Panico Hospital, Tricase (LE)), Francesco Giudici, MD (General Surgery and IBD Unit, Careggi University Hospital, Firenze), Fabio Cianchi, MD (General Surgery and IBD Unit, Careggi University Hospital, Firenze), Gabriele Baldini, MD (General Surgery and IBD Unit, Careggi University Hospital, Firenze), Ugo Pace, MD (Colorectal Surgical Oncology, Istituto Nazionale per lo Studio e la Cura dei Tumori, “Fondazione Giovanni Pascale IRCCS-Italia”, Napoli), Andrea Fares Bucci, MD (Colorectal Surgical Oncology, Istituto Nazionale per lo Studio e la Cura dei Tumori, “Fondazione Giovanni Pascale IRCCSItalia”, Napoli), Antonio Sciuto, MD (General Surgery Unit, ASL Napoli 2 Nord, Pozzuoli (NA)), Desirée Cianflocca, MD (General & Oncologic Surgery Unit, Department of Surgery, Santa Croce e Carle Hospital, Cuneo), Marco Migliore, MD (General & Oncologic Surgery Unit, Department of Surgery, Santa Croce e Carle Hospital, Cuneo), Michele Simone, MD (Department of Surgical Oncology, IRCCS Istituto Tumori “Giovanni Paolo II”, Bari), Marcella Lodovica Ricci, MD (Department of Surgery, Marche Nord Hospital, Pesaro e Fano (PU)), Francesco Monari, MD (General Surgery Unit, Infermi Hospital, Rimini), Alessandro Cardinali, MD (General Surgery Unit, S. Lucia Hospital, Macerata), Massimo Sartelli, MD (General Surgery Unit, S. Lucia Hospital, Macerata), Marcello Spampinato, MD, PhD, FEBS (HPB) (General Surgery Unit, “V. Fazzi” Hospital, Lecce), Alessandra Aprile, MD (General & Oncologic Surgery Unit, IRCCS “San Martino” National Cancer Center, Genova), Domenico Soriero, MD (General & Oncologic Surgery Unit, IRCCS “San Martino” National Cancer Center, Genova), Gabriella Teresa Capolupo, MD, FACS (Colorectal Surgery Unit, Policlinico Campus BioMedico, Roma), Jacopo Andreuccetti, MD (2nd General Surgery Unit 2, Spedali Civili di Brescia), Ilaria Canfora, MD (2nd General Surgery Unit 2, Spedali Civili di Brescia), Andrea Scarinci, MD (General Surgery Unit, Regina Apostolorum Hospital, Albano Laziale (RM)), Angela Maurizi, MD (General Surgery Unit, “C. Urbani” Hospital, Jesi (AN)), Grazia Maria Attinà, MD (General & Emergency Surgery Unit, San Camillo-Forlanini Hospital, Roma), Giulia Maggi, MD (Department of Gastrointestinal Surgery Unit, San Raffaele Research Hospital and “Vita-Salute” San Raffaele University, Milano), Umberto Bracale, MD (Minimally Invasive General and Oncologic and Surgery Unit, “Federico II” University, Napoli), Roberto Peltrini, MD (Minimally Invasive General and Oncologic and Surgery Unit, “Federico II” University, Napoli), Pietro Amodio, MD (General Oncologic Surgery Unit, Belcolle Hospital, Viterbo), Domenico Spoletini, MD, PhD, FACS (General Surgery Unit, S. Eugenio Hospital, ASL Roma 2), Rosa Marcellinaro, MD (General Surgery Unit, S. Eugenio Hospital, ASL Roma 2), Giovanni Del Vecchio, MD (General Surgery Unit, S. Carlo Hospital, Potenza), Massimo Stefanoni, MD (General Surgery Unit, Latisana-Palmanova Hospital, Friuli Centrale University (UD)), Carmelo Magistro, MD (General Oncologic and Mininvasive Surgery Unit, Great Metropolitan Niguarda Hospital, Milano), Diletta Cassini, MD (General Surgery Unit, ASST Ovest Milanese, Nuovo Ospedale di Legnano, Legnano (MI)), Lorenzo Crepaz, MD (General and Minimally Invasive Surgery Unit, San Camillo Hospital, Trento), Andrea Budassi, MD (General Surgery Unit, Profili Hospital, Fabriano (AN)), Bruno Sensi, MD (Minimally Invasive Surgery Unit, Policlinico Tor Vergata University Hospital, Roma), Silvia Tenconi, MD (General Surgery Unit, Gallarate Hospital (VA)), Leonardo Solaini, MD (General & Oncologic Surgery Unit, AUSL Romagna, Forlì (FC)), Giorgio Ercolani, MD (General & Oncologic Surgery Unit, AUSL Romagna, Forlì (FC)), Sarah Molfino, MD (General Surgery Unit 3, Department of Clinical and Experimental Sciences, University of Brescia), Giovanni Domenico De Palma, MD (General & Endoscopic Surgery Unit, “Federico II” University, Napoli), Paolo Locurto, MD (General Surgery Unit, S. Elia Hospital, Caltanissetta), Antonio Di Cintio, MD (General Surgery Unit, S. Maria Hospital, Terni), Lorenzo Pandolfini, MD (General Surgery Unit, Santa Maria Annunziata Hospital, Firenze), Alessandro Falsetto, MD (General Surgery Unit, Santa Maria Annunziata Hospital, Firenze), Elisa Cassinotti, MD (General Surgery Unit, Fondazione IRCCS Ca’ Granda, Policlinico Maggiore Hospital, Milano), Andrea Sagnotta, MD, PhD (General & Oncologic Surgery Unit, San Filippo Neri Hospital, ASL Roma 1), Monica Ortenzi, MD (Surgical Clinic, Torrette Hospital, University of Ancona), Alberto Biondi, MD (General Surgery Unit, Fondazione Policlinico Universitario Agostino Gemelli IRCCS, Roma), Giacomo Martorelli, MD (General Surgery Unit, “Ceccarini” Hospital, Riccione (RN)), Maurizio De Luca, MD (General Surgery Unit, S. Maria della Misericordia Hospital, Rovigo), Francesco Carrano, MD (Department of Biomedical Sciences, Humanitas University, Pieve Emanuele (MI) and IRCCS Humanitas Research Hospital, Rozzano (MI)), Annalisa Maroli, PhD (Department of Biomedical Sciences, Humanitas University, Pieve Emanuele (MI) and IRCCS Humanitas Research Hospital, Rozzano (MI)), Francesca Fior, MD (General Surgery Unit, University Hospital, Verona), Antonio Ferronetti, MD (General & Oncologic Surgery Unit, Evangelico Betania Hospital, Napoli), Giuseppe Giuliani, MD (General and Emergency Surgery Unit, Misericordia Hospital, Grosseto), Roberto Benigni, MD (General and Emergency Surgery Unit, Misericordia Hospital, Grosseto), Graziella Marino, MD (Abdominal Oncologic Surgery Unit, IRCCS CROB Basilicata Referral Cancer Center, Rionero in Vulture (PZ)), Patrizia Marsanic, MD (General Surgery Unit, “E. Agnelli” Hospital, Pinerolo (TO)), Nicoletta Sveva Pipitone Federico, MD (General Surgery Unit, “E. Agnelli” Hospital, Pinerolo (TO)), Carlo Di Marco, MD (General Surgery Unit, AULSS2 Marca Trevigiana, Conegliano Veneto (TV)), Camillo Leonardo Bertoglio, MD, PhD (General Surgery Unit, Fornaroli Hospital, ASST Ovest Milanese, Magenta (MI)), Francesca Pecchini, MD (General Surgery Unit, Civil Hospital, Baggiovara (MO)), Vincenzo Greco, MD (General Surgery Unit, Villa dei Gerani Hospital, Vibo Valentia (VV)), Michele Motter, MD (1st General Surgery Unit, S. Chiara Hospital, Trento), Giuseppe Tirone, MD (1st General Surgery Unit, S. Chiara Hospital, Trento), Marco Clementi, MD (General Surgery Unit, University of L’Aquila), Nicolò Tamini, MD (Colorectal Surgery Unit, San Gerardo Hospital, ASST Monza), Riccardo Piagnerelli, MD (General & oncologic Surgery Unit, AOU Senese, Siena), Giuseppe Canonico, MD (General Surgery Unit, San Giovanni di Dio Hospital, Firenze), Simone Cicconi, MD (General Surgery Unit, “C. e G. Mazzoni” Hospital, Ascoli Piceno), Marco Colasanti, MD (General & Transplant Surgery Unit, San Camillo-Forlanini Hospital, Roma), Enrico Pinotti, MD (General & Mininvasive Surgery Unit, S. Pietro Hospital, Ponte San Pietro (BG)), Roberta Carminati, MD (General Surgery Unit, Pesenti Fenaroli Hospital, Alzano Lombardo (BG)), Edoardo Osenda, MD (Surgical Clinic, University of Trieste), Luigina Graziosi, MD (General & Emergency Surgery Unit, University of Perugia), Ciro De Martino, MD (General & Emergency Surgery Unit, S. Giovanni di Dio e Ruggi d’Aragona Hospital, Salerno), Giovanna Ioia, MD (General & Emergency Surgery Unit, S. Giovanni di Dio e Ruggi d’Aragona Hospital, Salerno), Arianna Birindelli, MD (General Surgery Unit, ASST Valcamonica, Esine (BS)), Matteo Chiozza, MD (General & Laparoscopic Surgery Unit, University Hospital, Ferrara), Daniele Zigiotto, MD (General & Upper GI Surgery Unit, University Hospital, Verona), Fioralba Pindozzi, MD (General Surgery Unit, Delta Hospital, Lagosanto (FE)), Manuela Grivon, MD (General Surgery Unit, “U. Parini” Regional Hospital, Aosta), Cristian Conti, MD (General & HPB Surgery Unit, University Hospital, Verona), Lorenzo Organetti, MD (General Surgery Unit, “A. Murri” Hospital, Fermo), Michela Monteleone, MD (General Surgery Unit, S. Leopoldo Hospital, Merate (LC)), Emanuele Botteri, MD (General Surgery Unit, Spedali Civili of Brescia, Montichiari (BS)), Giorgio Dalmonte, MD (Surgical Clinic, University of Parma), Diletta Frazzini, MD (General Surgery Unit, Spirito Santo Hospital, Pescara), Simone Santoni, MD (General Surgery Unit, Policlinico Casilino, Roma), Gabriele La Gioia, MD (General Surgery Unit, S. Maria della Misericordia Hospital, Urbino (PU)), Diana Giannarelli, MS, PhD (Clinical Trial Center, Biostatistics and Bioinformatics, Fondazione Policlinico Universitario Agostino Gemelli IRCCS, Roma, Italy).

## Supplementary Material


