# Supplementary material for: Patient-Reported Outcomes and Return to Intended Oncologic Therapy After Colorectal Enhanced Recovery Pathway: The iCral3 Prospective Study
Source: Ann Surg Open. 2023 Mar 8;4(1):e267. doi: 10.1097/AS9.0000000000000267 (PMC10431437; doi:10.1097/AS9.0000000000000267)
Supplement: Supplementary file 1 [file as9-4-e267-s001.pdf]

Suppl. Tab. 1: Study variables patterns and frequencies in the population analyzed for patient-reported outocome measures (PROMs) and in the population analyzed for the return to intended oncologic therapy (RIOT).

		PROMs population No. = 4,529		RIOT populaton No. = 1,467	
**Variable**	**Pattern**	**No.**	**%**	**No.**	**%**
Age (years)	≤ 69	2,308	51.0	755	51.5
	> 69	2,221	49.0	712	48.5
Gender	Female	2,140	47.2	630	42.9
	Male	2,389	52.8	837	57.1
ASA Class	I-II	2,992	66.1	959	65.4
	III	1,537	33.9	508	34.6
Body Mass Index (Kg/m^2)	≤ 25.0	2,190	48.4	689	47.0
	25.1-30.0	1,719	37.9	571	38.9
	> 30.0	620	13.7	207	14.1
Diabetes	Yes	630	13.9	211	14.4
	No	3,899	86.1	1,256	85.6
Chronic renal failure	Yes	195	4.3	60	4.1
	No	4,334	95.7	1,407	95.9
Dialysis	Yes	10	0.2	3	0.2
	No	4,519	99.8	1,464	99.8
Perioperative steroids	Yes	79	1.7	16	1.1
	No	4,450	98.3	1,451	98.9
Neoadjuvant therapy	Yes	336	7.4	183	12.5
	No	4,193	92.6	1,284	87.5
Chronic liver disease	Yes	52	1.1	20	1.4
	No	4,477	98.9	1,447	98.6
MNA-SF	< 12	1,581	34.9	549	37.4
	≥ 12	2,948	65.1	918	62.6
Preoperative blood transfusions	Yes	273	6.0	108	7.4
	No	4,256	94.0	1,359	92.6
Intra- and postoperative blood transfusions	Yes	304	6.7	111	7.6
	No	4,225	93.3	1,356	92.4
Center volume (no. of enrolled cases)	low (≤ 44)	1,023	22.6	355	24.2
	high (> 44)	3,506	77.4	1,112	75.8
Institutional ERP	Yes	3,001	66.3	939	64.0
	No	1,528	33.7	528	36.0
Hospital type	District/regional	2,086	46.1	635	43.3
	Academic/teaching	1,423	31.4	503	34.3
	Metropolitan	1,020	22.5	329	22.4
Surgical Unit type	General surgery	3,826	84.5	1,218	83.0
	Oncologic surgery	299	6.6	142	9.7
	Colorectal surgery	404	8.9	107	7.3
Admission	Elective	4,236	93.5	1,368	93.3
	Delayed urgency	293	6.5	99	6.7
Procedure	Right colectomy	1,664	36.7	573	39.0

--

	Transverse colectomy	91	2.0	35	2.4
	Splenic flexure colectomy	133	2.9	60	4.1
	Left colectomy	1,152	25.4	322	21.9
	Anterior resection	1,001	22.1	395	26.9
	TA-TME	51	1.1	24	1.6
	Hartmann reversal	111	2.5	8	0.5
	(Sub)total colectomy	87	1.9	18	1.2
	Other	239	5.3	32	2.2
Procedure 2	Standard	3,817	84.3	1,290	87.9
	Non-standard	712	15.7	177	12.1
Approach	Laparoscopic	3,138	69.3	953	65.0
	Robotic	508	11.2	193	13.1
	Converted	253	5.6	103	7.0
	Open	630	13.9	218	14.9
Procedure length (minutes)	≤ 180	2,375	52.4	741	50.5
	> 180	2,154	47.6	726	49.5
Indication to surgery	Cancer	3,283	72.5	1,467	100.0
	Polyp(s)	230	5.1	--	--
	Diverticular disease	504	11.1	--	--
	Endometriosis	165	3.7	--	--
	IBD	155	3.4	--	--
	Other	192	4.2	--	--
Associated procedures	No	3,696	81.6	1,194	81.4
	Yes	833	18.4	273	18.6
	Cholecystectomy	122	--	33	--
	Small bowel resection	45	--	22	--
	Adnexectomy	37	--	17	--
	Bladder resection	16	--	4	--
	Minor liver resection	79	--	64	--
	Major liver resection	22	--	12	--
	Hysterectomy	14	--	5	--
	Other colorectal resection	41	--	15	--
	Partial duodenectomy	9	--	8	--
	Splenectomy	5	--	1	--
	Other	443	--	92	--
ERP adherence (%)	≤ 69.2	2,520	55.6	808	55.1
	> 69.2	2,009	44.4	659	44.9
ERP adherence centile (%)	1st (≤ 53.8)	1,303	28.8	365	24.9
	2nd (53.9-69.2)	1,217	26.9	443	30.2
	3rd (69.3-80.8)	1,131	25.0	380	25.9
	4th (80.8)	878	19.3	279	19.0
Nutritional screening	Yes	3,974	87.7	1,296	88,3
	No	555	12.3	171	11.7
Nutritional prehabilitation	Yes	1,469	32.4	476	32.4
	No	3,060	67.6	991	67.6
Physical prehabilitation	Yes	1,058	23.4	335	22.9
	No	3,471	76.6	1,132	77.1

Psychological prehabilitation	Yes	812	17.9	271	18.5
	No	3,717	82.1	1,196	81.5
Counseling	Yes	3,079	68.0	1,088	74.2
	No	1,450	32.0	379	25.8
Preoperative IEN	Yes	1,418	31.3	508	34.6
	No	3,111	68.7	959	65.4
Preoperative anemia screening-correction	Yes	552	12.2	209	14.2
	No	3,977	87.8	1,258	85.8
DVT-PE prophylaxis	Yes	4,160	91.8	1,342	91.5
	No	369	8.2	125	8.5
Antibiotic prophylaxis	Yes	4,355	96.2	1,419	96.7
	No	174	3.8	48	3.3
No mechanical bowel preparation	Yes	2,807	62.0	925	63.1
	No	1,722	38.0	542	36.9
Preoperative carbohydrates load	Yes	2,527	55.8	849	57.9
	No	2,002	44.2	618	42.1
Preoperative 2-6 hours fasting	Yes	3,248	71.7	1,089	74.2
	No	1,281	28.3	378	25.8
No preanesthesia	Yes	3,296	72.8	1,107	75.4
	No	1,233	27.2	360	24.6
Standard anesthesia protocol	Yes	3,715	82.0	1,254	85.5
	No	814	18.0	213	14.5
Normothermia	Yes	4,098	90.5	1,327	90.5
	No	431	9.5	140	9.5
Restrictive or goal-directed fluid therapy	Yes	3,207	70.8	1,085	74.0
	No	1,322	29.2	382	26.0
PONV prophylaxis	Yes	4,016	88.7	1,306	89.0
	No	513	11.3	161	11.0
Multimodal analgesia	Yes	4,054	89.5	1,319	89.9
	No	475	10.5	148	10.1
Minimally invasive surgery	Yes	3,899	86.1	1,236	84.2
	No	630	13.9	231	15.8
No nasogastric tube	Yes	3,708	81.9	1,173	80.0
	No	821	18.1	294	20.0
No postoperative major opiates	Yes	3,429	75.7	1,100	75.0
	No	1,100	24.3	367	25.0
No abdominal drain(s)	Yes	3,068	67.7	480	32.7
	No	1,461	32.3	987	67.3
Urinary cathether removed at 24-48 hours	Yes	3,287	72.6	1,102	75.1
	No	1,242	27.4	365	24.9
Early postoperative mobilization	Yes	2,542	56.1	815	55.6
	No	1,987	43.9	652	44.4
Early postoperative feeding	Yes	2,579	56.9	806	54.9
	No	1,950	43.1	661	45.1
Pre-discharge check	Yes	3,540	78.2	1,206	82.2
	No	989	21.8	261	17.8

Anastomotic leakage	Yes	205	4.5	67	4.6
	No	4,324	95.5	1,400	95.4
Overall Morbidity	Yes	1,214	26.8	396	27.0
	No	3,315	73.2	1,071	73.0
Major morbidity	Yes	341	7.5	122	8.3
	No	4,188	92.5	1,345	91.7
Reoperation	Yes	232	5.1	77	5.2
	No	4,297	94.9	1,390	94.8

ASA: American Society of Anesthesiologists; MNA-SF: mini nutritional assessment short form; TA-TME: Transanal total mesorectal excision; IBD: Inflammatory bowel disease; IEN: Immune enhancing nutrition; DVT-PE: deep venous thrombosis/pulmonary embolism; PONV: postoperative nausea/vomiting;
