# Supplementary material for: Patient-Reported Outcomes and Return to Intended Oncologic Therapy After Colorectal Enhanced Recovery Pathway: The iCral3 Prospective Study
Source: Ann Surg Open. 2023 Mar 8;4(1):e267. doi: 10.1097/AS9.0000000000000267 (PMC10431437; doi:10.1097/AS9.0000000000000267)
Supplement: Supplementary file 2 [file as9-4-e267-s002.pdf]

Suppl. Tab. 2: Raw (unadjusted) PROMs values.

	Valid Data (%)	Mean ± SD	95% CI	Median	IQR	Range	p
**EQ-5D-5L**							
preoperative	4,248 (93.8)	92.7 ± 20.5	92.1-93.4	95	82-105	5-125	
discharge	4,238 (93.6)	89.0 ± 19.5	88.4-89.6	92	79-103	6-125	< .0001
late	4,084 (90.2)	101.3 ± 18.3	100.8-101.9	105	93-115	5-125	
**MDASI-GI**							
preoperative	4,324 (95.5)	39.0 ± 35.7	38.0-40.1	29	12-56	0-217	
discharge	4,223 (93.2)	38.2 ± 31.9	37.2-39.1	30	14-53	0-192	< .0001
late	4,066 (89.8)	20.7 ± 24.8	20.0-21.5	12	4-28	0-163	
**FACT-C**							
preoperative	4,090 (90.3)	95.7 ± 16.5	95.2-96.2	98	86-107	4-136	
discharge	4,068 (89.8)	94.6 ± 15.0	94.1-95.0	97	85-105	24-136	< .0001
late	3,990 (88.1)	100.5 ± 18.4	99.9-101.0	104	94-112	7-136	

SD: standard deviation; IQR: interquartile range; EQ-5D-5L: Euro-Quality of Life Group EQ-5D-5L; MDASI-GI: MD Anderson Symptom Inventory for Gastrointestinal Surgery patients; FACT-C: Functional Assessment of Cancer Therapy – Colorectal.
