# Supplementary material for: Patient-Reported Outcomes and Return to Intended Oncologic Therapy After Colorectal Enhanced Recovery Pathway: The iCral3 Prospective Study
Source: Ann Surg Open. 2023 Mar 8;4(1):e267. doi: 10.1097/AS9.0000000000000267 (PMC10431437; doi:10.1097/AS9.0000000000000267)
Supplement: Supplementary file 3 [file as9-4-e267-s003.pdf]

	EQ-5D-5L						MDASI-GI						FACT-C					
	Discharge			Late			Discharge			Late			Discharge			Late		
**Variable**	**Beta**	**SE**	**p**	**Beta**	**SE**	**p**	**Beta**	**SE**	**p**	**Beta**	**SE**	**p**	**Beta**	**SE**	**p**	**Beta**	**SE**	**p**
(Intercept)	46.84	4.38	<.001	74.10	3.45	<.001	16.70	4.12	<.001	4.04	3.08	.191	37.11	3.80	<.001	56.82	7.10	<.001
Age > 69 years	0.34	0.69	.626	-1.58	0.47	<.001	-3.61	1.25	.004	-0.82	0.95	.386	0.21	0.44	.638	-0.42	0.71	.559
Male sex	2.26	0.54	<.001	0.52	0.63	.404	-2.64	0.92	.004	-1.80	0.73	.014	0.56	0.44	.204	0.46	0.58	.426
ASA Class III	-1.94	0.71	.007	-2.86	0.65	<.001	1.46	1.08	.176	3.19	0.92	<.001	-1.64	0.63	.010	-1.76	0.81	.031
BMI > 30.0 Kg/m^2	1.10	0.83	.184	-0.27	0.77	.731	-0.97	1.15	.401	0.09	0.93	.920	1.04	0.48	.029	-0.66	0.91	.468
BMI 25.1-30.0 Kg/m^2	0.89	0.65	.170	0.08	0.48	.864	-1.67	0.87	.056	1.05	0.73	.151	0.10	0.40	.810	-0.95	0.69	.168
MNA-SF ≥ 12	0.19	1.12	.865	0.17	0.98	.859	-0.45	2.06	.827	0.34	1.42	.809	1.63	0.71	.022	0.03	1.17	.976
Diabetes	-1.41	0.98	.149	-0.60	0.82	.486	0.54	1.44	.709	0.89	1.08	.410	-0.54	0.62	.382	-1.32	0.86	.128
Chronic renal failure	-2.66	1.22	.030	-4.04	1.34	.003	2.79	2.36	.236	2.95	1.72	.086	-2.66	1.02	.009	-2.67	1.46	.067
Perioperative steroids	0.59	2.54	.818	-1.39	1.99	.486	-2.40	3.62	.508	1.64	2.64	.533	0.33	1.31	.803	0.57	1.81	.753
Neoadjuvant therapy	-4.01	1.14	<.001	-4.82	1.07	<.001	3.68	1.89	.052	6.08	1.33	<.001	-2.07	0.70	.003	-2.64	0.96	.006
Chronic liver disease	1.25	2.08	.546	-0.46	1.91	.809	-3.90	4.50	.385	0.45	2.58	.863	3.72	1.71	.030	-1.15	1.92	.549
Delayed urgency admission	1.80	2.29	.432	0.04	2.06	.985	-7.55	3.05	.013	-2.58	1.95	.187	4.19	1.52	.006	3.70	2.01	.066
Preoperative blood transfusion(s)	-0.88	1.05	.405	-2.03	1.10	.065	-0.76	1.45	.599	1.72	1.62	.288	1.13	0.86	.189	1.53	1.32	.169
Intra-postoperative blood transfusion(s)	-2.25	1.28	.079	-0.39	1.30	.761	2.00	1.99	.314	1.14	1.61	.478	-0.52	1.00	.602	0.16	1.20	.892
Procedure length > 180 minutes	-2.12	0.86	.013	-1.23	0.69	.075	4.41	1.45	.002	2.45	1.02	.017	-1.17	0.60	.052	-0.76	0.70	.280
Associated procedures	-2.82	1.54	.067	-1.34	1.79	.454	5.00	2.66	.060	1.41	1.69	.404	-1.39	0.89	.117	-1.81	1.31	.169
Minimally invasive surgery	-0.61	1.35	.654	0.73	0.99	.462	2.60	2.30	.258	0.32	1.56	.837	0.47	0.90	.603	0.74	1.11	.506
ERP adherence rate 4th centile	-1.48	1.68	.377	-0.84	1.31	.522	-0.30	2.64	.910	-1.05	1.46	.472	0.21	1.20	.864	0.42	1.89	.824
High volume center	1.00	1.58	.529	-0.02	1.23	.987	-5.03	3.33	.131	-1.77	2.12	.404	2.18	1.07	.043	0.59	1.77	.739
Institutional ERP center	-0.90	2.08	.664	-2.57	1.60	.109	1.30	4.80	.786	-1.20	2.57	.640	-1.86	1.32	.159	-1.93	1.72	.264
Anastomotic leakage	-6.50	2.00	.001	-4.76	1.44	<.001	7.49	3.95	.058	4.44	2.51	.077	-4.55	1.36	<.001	-5.01	1.79	.005
Overall morbidity	-1.62	0.68	.018	-1.42	0.55	.010	2.95	1.15	.011	1.73	0.74	.020	-0.82	0.48	.090	-1.11	0.61	.070
Major morbidity	-1.71	2.04	.403	-2.27	1.36	.095	-0.64	2.87	.823	3.02	2.84	.287	-1.44	1.30	.268	-2.28	1.88	.226
Reoperation	-3.72	2.58	.151	-2.04	2.04	.317	7.18	3.73	.054	1.49	3.86	.701	-0.77	1.66	.644	2.07	1.90	.278

Standard procedure	-2.56	1.08	.018	-0.03	0.84	.975	2.48	1.65	.133	0.07	1.05	.950	-0.67	0.76	.380	0.77	0.99	.436
Surgery for malignancy	0.80	1.15	.489	-3.69	1.24	.003	-0.62	2.08	.766	3.70	1.55	.017	-0.36	0.60	.552	-3.47	1.00	<.001
PROMs preoperative values	0.50	0.04	<.001	0.38	0.04	<.001	0.50	0.04	<.001	0.33	0.03	<.001	0.61	0.03	<.001	0.50	0.07	<.001
(Scale)	256.00			248.67			665.42			457.95			126.35			258.96		

SE: standard error; ASA: American Society of Anesthesiologists; BMI: body mass index; BMI: body mass index; MNA-SF: mini nutritional assessment short form; ERP: enhanced recovery pathway; EQ-5D-5L: Euro-Quality of Life Group EQ-5D-5L; MDASI-GI: MD Anderson Symptom Inventory for Gastrointestinal Surgery patients; FACT-C: Functional Assessment of Cancer Therapy – Colorectal.

Suppl. Tab.3: Regression coefficients after a generalized linear mixed model considering 4th centile of ERP adherence rates;
